# Antioxidant, Antimicrobial and Antiviral Properties of Herbal Materials

**DOI:** 10.3390/antiox9121309

**Published:** 2020-12-21

**Authors:** Shokoh Parham, Anousheh Zargar Kharazi, Hamid Reza Bakhsheshi-Rad, Hadi Nur, Ahmad Fauzi Ismail, Safian Sharif, Seeram RamaKrishna, Filippo Berto

**Affiliations:** 1 Department of Biomaterials, Nanotechnology and Tissue Engineering, Faculty of Advanced Technologies in Medicine, Isfahan University of Medical Sciences, Isfahan 8174673461, Iran; a_zargar@med.mui.ac.ir; 2 Advanced Materials Research Center, Department of Materials Engineering, Najafabad Branch, Islamic Azad University, Najafabad, Iran; 3 Faculty of Engineering, Universiti Teknologi Malaysia, Johor Bahru, Johor 81310, Malaysia; safian@utm.my; 4 Centre for Sustainable Nanomaterials, Ibnu Sina Institute for Scientific and Industrial Research, Universiti Teknologi Malaysia, UTM Skudai, Johor 81310, Malaysia; hadi@kimia.fs.utm.my; 5 Advanced Membrane Technology Research Center (AMTEC), Universiti Teknologi Malaysia, Johor Bahru, Johor 81310, Malaysia; afauzi@utm.my; 6 Department of Mechanical Engineering, National University of Singapore, 9 Engineering Drive 1, Singapore 117576, Singapore; 7 Department of Mechanical and Industrial Engineering, Norwegian University of Science and Technology, 7491 Trondheim, Norway

**Keywords:** herbal material, reactive oxygen species (ROS), antimicrobial, antioxidant, medicine applications, antiviral, virucidal

## Abstract

Recently, increasing public concern about hygiene has been driving many studies to investigate antimicrobial and antiviral agents. However, the use of any antimicrobial agents must be limited due to their possible toxic or harmful effects. In recent years, due to previous antibiotics’ lesser side effects, the use of herbal materials instead of synthetic or chemical drugs is increasing. Herbal materials are found in medicines. Herbs can be used in the form of plant extracts or as their active components. Furthermore, most of the world’s populations used herbal materials due to their strong antimicrobial properties and primary healthcare benefits. For example, herbs are an excellent material to replace nanosilver as an antibiotic and antiviral agent. The use of nanosilver involves an ROS-mediated mechanism that might lead to oxidative stress-related cancer, cytotoxicity, and heart diseases. Oxidative stress further leads to increased ROS production and also delays the cellular processes involved in wound healing. Therefore, existing antibiotic drugs can be replaced with biomaterials such as herbal medicine with high antimicrobial, antiviral, and antioxidant activity. This review paper highlights the antibacterial, antiviral, and radical scavenger (antioxidant) properties of herbal materials. Antimicrobial activity, radical scavenger ability, the potential for antimicrobial, antiviral, and anticancer agents, and efficacy in eliminating bacteria and viruses and scavenging free radicals in herbal materials are discussed in this review. The presented herbal antimicrobial agents in this review include clove, portulaca, tribulus, eryngium, cinnamon, turmeric, ginger, thyme, pennyroyal, mint, fennel, chamomile, burdock, eucalyptus, primrose, lemon balm, mallow, and garlic, which are all summarized.

## 1. Introduction

**Figure 1:**
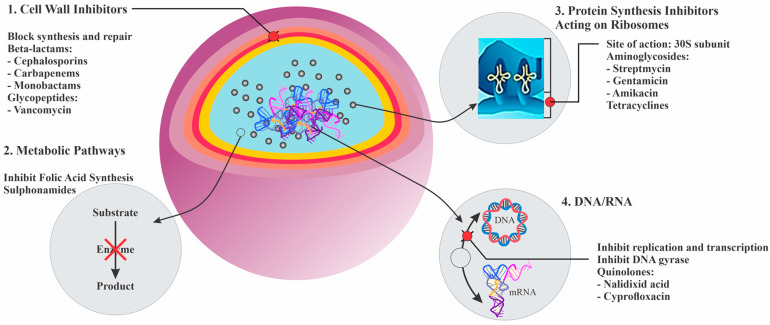
Illustration of the various antimicrobial mechanisms of antibiotics that interrupt bacterial cells [13,14].

**Figure 2:**
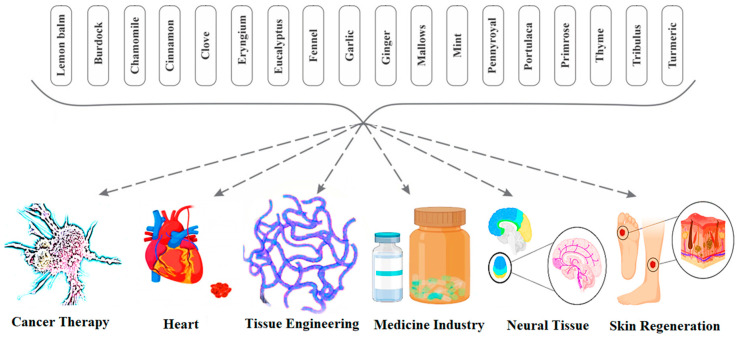
The biomedical applications of herbal materials [18].

Due to increasing concerns about the sustainability of human living, the control of the damaging effects of microorganisms is becoming very important. A wide range of microorganisms exist in a biological balance with the human body and its living environments, but an uncontrolled and rapid growth of microbes can lead to some dangerous problems [1,2,3,4]. Antimicrobial agents are used as antibiotic drugs to control infections in the human body, but they can cause many side effects, especially increasing reactive oxygen species (ROS) in the human body [5,6]. ROS are very dangerous to human health and well-being and play a role in producing cancer [7,8,9]; further, they can increase potential health risks [10,11,12]. Figure 1 demonstrates the different antimicrobial mechanisms of antibiotics for disrupting bacterial cells [13,14]. The herbal materials used as medicinal plants include several types of plants. Many of these herbal materials show medicinal activities such as antioxidant, anticancer, anti-inflammatory, antimicrobial, and antiviral activities. Furthermore, these herbs can play the main role in drug synthesis and development. These materials show a significant role in different biological applications such as cancer therapy, cardiovascular disease treatment, neural disease treatment and skin regeneration [15,16,17]. The biomedical applications of these materials are illustrated in Figure 2 [18]. Herbal medicines performed the primary medicinal functions in ancient cultures in Africa, Europe, the Americas, and especially in Asia [19,20,21]. Herbal medicines are the primary medicine to treat infection in some developing countries. The extracts of herbal materials signify continuous attempts to investigate new compounds with potential antibacterial activity [22]. Several studies have shown that different herbal medicines are sources of diverse molecules, many of which exhibit radical scavenger and antimicrobial properties which can defend the human body against pathogens and also cellular oxidation reactions. Therefore, these materials are significant in synthesizing different types of herbal medicine for their antimicrobial, antiviral, and antioxidant potential [23,24,25,26,27]. These diverse molecules can control and inhibit pathogens with low toxicity to cells and are therefore considered as materials for new antimicrobial medicine research. Based on those above control methods for the terrible effect of the rapid growth of bacteria and viruses, many studies have focused on researching antibacterial and antiviral medicines with lower side effects [28,29,30,31].

## 2. Antimicrobial Agent

**Figure 3:**
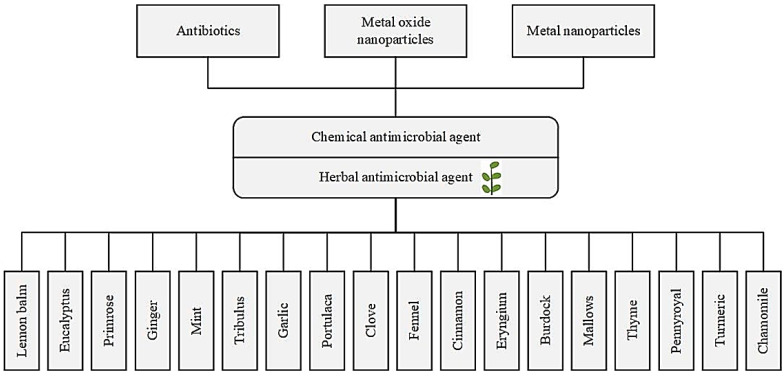
The antimicrobial agents’ classification based on their different substances.

An agent which can kill microorganisms or stop their growth is known as an antimicrobial agent or antimicrobial medicine. Antimicrobial medicines are categorized based on the primary microorganisms they act against such as bacteria and viruses [32]. Antimicrobial agents are divided into two groups based on their different chemical substances. The first group is synthetic antimicrobial agents (chemical antimicrobial agents) including antibiotic drugs and metal and metal oxide nanoparticles including silver, silver oxide, and so on. The second group is herbal antimicrobial agents. Figure 3 shows the classification of the different antimicrobial agents based on their chemical substances.

1. Antibiotics and other chemical antimicrobial agents play a big role as antimicrobial agents, but they lead to various side effects. One of the main side effects is the generation of free oxygen radicals (ROS). ROS are very toxic and have been thought to play a main role in producing cancer [8,12,13].
2. The second group is related to herbal antimicrobial agents, such as clove, portulaca, tribulus, eryngium, cinnamon, turmeric, ginger, thyme, pennyroyal, mint, fennel, chamomile, burdock, eucalyptus, primrose, lemon balm, mallows, and garlic. These biomaterials can act as free radical scavengers and can therefore block the production of ROS. A brief list of essential biomaterials that can act as antimicrobial agents without any toxicity is shown in Table 1.

**Figure 4:**
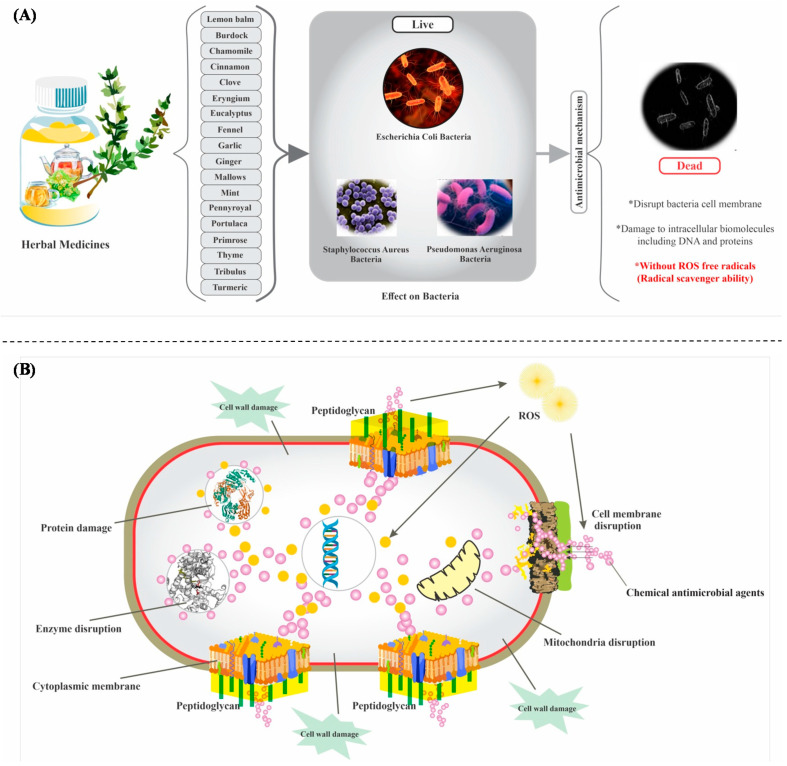
The antimicrobial mechanisms of herbal antimicrobial agents (**A**) against chemical antimicrobial agents (**B**) [34,35].

Recently, using the natural material has become ideal for treatment of microbial infections due to the possible toxic or harmful effects of many chemical antimicrobial agents [33]. Figure 4 shows the antimicrobial mechanisms of chemical antimicrobial agents against herbal antimicrobial agents [34,35]. The application of herbal materials would be an ideal alternative [36,37] and can also open up a new chance for producing new anticancer, antimicrobial, and antiviral drugs with lower side effects. There is much research that has demonstrated this material as a potential antimicrobial agent. This review paper is concerned with the pharmacological effects and properties of several herbal plants including clove, portulaca, tribulus, eryngium, cinnamon, turmeric, ginger, thyme, pennyroyal, mint, fennel, chamomile, burdock, eucalyptus, primrose, lemon balm, mallows, and garlic with good potential for antimicrobial, antiviral, and radical scavenger abilities.

### 2.1. Clove

Clove (*Syzygium aromaticum)*, from the *Myrtaceae* family, is one of the most effective antimicrobial and antioxidant herbs. This herb is one of the traditional herbs primarily local to Asia and Africa. Based on the bioactive components of clove such as eugenol, eugenyl acetate, α-humulene, 2-heptanone, and β-caryophyllene, it can display many pharmacological activities such as antimicrobial, antioxidant, anti-inflammatory, antimutagenic, anticancer, and anti-allergic properties. These bioactive components allow clove to demonstrate one of the highest potent antioxidant activities among other herbal medicines. Previous studies have reported the sufficient antibacterial property of clove extract and oil against different strains of bacteria (Gram-positive and Gram-negative) [39,72]. Other researchers have shown that clove has antimicrobial activity against many bacteria including *Listeria monocytogenes*, *Klebsiella pneumoniae*, *S. aureus*, *E. coli* and *S. Typhimurium* [73,74,75]. Clove based on eugenol plays an antimicrobial role. The antimicrobial mechanism of action of eugenol is that, at first, the eugenol molecule with high solubility can participate in the cytoplasmic membrane. Then, it creates disturbance as a consequence of its OH group. Finally, it can pass through the hydrophilic proportion of the cell. After that, the OH group of eugenol can bind to proteins of the membrane of bacteria and can permeate the fundamental cell components [76,77]. Clove extract with various amounts (1 and 3 mg mL^−1^) have been demonstrated to experience a great antimicrobial impact on *S. typhi* and *E. coli* [72]. Clove has strong antioxidant effects that can naturalize ROS and other free radicals in lipid chains. Therefore, they inhibit further oxidation of lipids [78]. Researchers have also observed that clove’s extract could inhibit the malondialdehyde formation from horse blood plasma oxidation [79]. Other researchers have reported the maximum antioxidant activity of clove against DPPH (2, 2-diphenyl-1-picryl hydracyl), than BHA (butylated hydroxyanisole), and BHT (butylated hydroxytoluene) radicals [80]. Similarly, the result of one research has shown the antioxidant activity of eugenol of clove extract against DPPH, ABTS and superoxide radicals [81]. Other studies have reported strong antioxidant activity of this herb against DPPH when compared to vitamin C [82]. The antiviral activity of this plant has been reported against different viruses such as herpes adenovirus, poliovirus, and coxsackievirus [83]. The high antioxidant activity of clove’s extract and essential oil is related to the chemical content of this herb, like a phenolic compound.

### 2.2. Portulaca

Portulaca is one of the traditional herbs from Asia. It has been reported to have potent antimicrobial and antioxidant activity. Several researchers have declared the biomedicine activities of portulaca over the past decades. The antioxidant effects of portulaca are the main factor of the biomedicine activity of this plant. Therefore, this plant can naturalize free radicals such as ROS in lipid chains. Hence, it can inhibit the further oxidation of lipids [40]. The antioxidant and antimicrobial property of portulaca is related to its components such as ascorbic acid, a-tocopherols, omega-3 fatty acids, apigenin, gallotannins, quercetin, and kaempferol. The antioxidant activity of portulaca is primarily related to omega-3 fatty acids [40,41]. Previous studies have shown the antimicrobial effects of this plant against different bacteria and fungi [84]. Furthermore, the pectic polysaccharide of this plant has been shown to have high antiviral properties against spatial viruses such as simplex virus type II [85]. Portulaca can be shown antibacterial activity against different bacteria (Gram-positive and Gram-negative) such as *Pseudomonas aeruginosa*, *Neisseria gonorrhea*, *E. coli (Escherichia coli)*, *Streptococcus faecalis*, *Bacillus* and *S. aureus (Staphylococcus aureus)* [86,87]. Researchers in the last decade have found that portulaca’s extracts demonstrate inhibitory ability against different bacteria (Gram-negative and Gram-positive). Other studies have reported the antifungal activity of portulaca extracts against various fungi using an automatic single-cell bioassay system. The antifungal activity of the portulaca was also revealed against fungi such as *Aspergillus yeast Candida* and *Trichophyton*. The ethylacetate extract of portulaca shows positive activity against dermatophytes of the genera *Trichophyton* [88]. The ethanol extracted of this plant has also been tested against *Bacillus* [89]. The antifungal activity of organic and aqueous solvent extracts of this medical plant is also reported against *Fusarium*, *Aspergillus niger*, and *Rhizopus artocarpi* [90]. The antioxidant and antimicrobial activity of this medicinal plant is based on its chemical content including phenolic compounds [40,41].

### 2.3. Tribulus

Tribulus (*Tribulus terrestris*) is from the *Zygophyllaceae* family. This herb is native to Southern Asia, Europe, and Africa [91]. This plant has shown several pharmacological activities including antioxidant, cardiotonic, antimicrobial, antihypertensive, anticancer, and analgesic characteristics [92,93]. The Iranian and Turkish tribulus has been shown to have high antibacterial activity [94,95]. The antimicrobial activity of different extracts from tribulus was examined against eleven microorganisms including *E. coli*, *S. aureus*, *Bacillus cereus*, *Corynebacterium diphtheria*, *Salmonella typhimurium*, *Candida albicans*, *Proteus vulgaris*, *K. pneumonia (Klebsiella pneumonia)*, *S. marcescens (Serratia marcescens)*, and *Pseudomonas aeruginosa*. Previous studies have found that all types of tribulus extracts such as chloroform and ethanol extracts demonstrate high antimicrobial properties against various microorganisms [96,97,98]. Tribulus extract has been investigated for its antioxidant activity, radical scavenging, and its ability to strongly inhibit lipid peroxidation. Past studies have reported that this herb can play the primary role as an authoritative natural source of antioxidants. Therefore, it can be useful in inhibiting pathologies of free radicals. Previous studies have investigated the extract of tribulus as a high source of flavonoids [42,99]. Furthermore, it is well known that total polyphenols including flavonoid, tannin, and phenolic acids are strongly related to the antioxidant activity [43,100,101,102,103,104]. This plant also shows inhibitory activity against HIV virus [105].

### 2.4. Eryngium

Eryngium (*Eryngium*) is one of the plants native to Central and Southeast Europe, America, and Central Asia. This plant belongs to the *Apiaceae* family. The different components of eryngium include flavonoids, phenolic acids, and coumarins which are key factors of the pharmacological property of this plant [106,107,108,109,110]. Based on the presence of this component, eryngium shows high antimicrobial and antioxidant activity. Previous studies have proved that the high antioxidant activity of eryngium is due to the presence of flavonoids in the plant extract [111]. Therefore, eryngium is shown to have some pharmacological activity such as antimicrobial, antioxidant, antidiuretic, antitussive, aphrodisiac, appetizer, and stimulant characteristics [44,112,113]. Some researchers have reported that this plant shows antibacterial, anti-yeast, antiviral, and antifungal activity, while the results also indicate that polyacetylenes of this plant demonstrated antifungal and antibacterial abilities. The extracts and essential oil of this plant show high antibacterial properties against *S. aureus, Listeria monocyatogenes, Bacillus, E. coli, Salmonella typhimurium, P. acnes., S. bovis, S. pyogenes, S. dysgalactiae, S. pneumonia*, and *Pseudomonas* [114,115,116,117].

### 2.5. Cinnamon

Cinnamon (*Cinnamomum verum* and *Cinnamomum zeylanicum*) is one of the plants that belong to the *Lauraceae* family. This traditional herbal medicine is from Australia and Asia [118,119,120]. Based on the antioxidant, antimicrobial, and anticarcinogenic activities of this plant, it is widely used in medical industries [120,121,122,123]. Previous investigations have found cinnamon to have antimicrobial characteristics [124,125,126,127]. Cinnamon has been traditionally used for its antiseptic, antioxidant, and antimicrobial properties. Previous studies have investigated the antimicrobial activities of cinnamon against various bacteria, such as *Bacillus* and *E. coli* [128]. Cinnamon oil has shown antibacterial effects against *E. coli, Listeria monocytogenes, Bacillus, Enterococcus faecalis, Salmonella typhimurium, Pseudomonas aeruginosa, Yersinia enterocolitica* and *Staphylococcus aureus* [129,130,131,132]. This strong antimicrobial activity is based on the presence of cinnamaldehyde and eugenol in cinnamon essential oil. Bacteria such as *Campylobacter jejuni* have been shown to be more inhibited by the essential oil of cinnamon compared to other Gram-negative bacteria such as *Escherichia coli* [47]. Other researchers have demonstrated the mechanism of the antimicrobial action of the essential oil of cinnamon against cell walls of *Listeria monocytogenes, E. coli*, and *S. aureus* [133,134,135]. The appearance of phenolic substances in cinnamon results in potential antioxidant, antimutagenic, antidiabetic, anticancer, and anti-inflammatory activities. The essential oil of this herb has been shown to have antioxidant activity. Other studies have reported antioxidant activities of water, methanol, and ethanolic cinnamon extracts [45,46,136,137,138]. This plant shows high anti-influenza virus activity [139].

### 2.6. Turmeric

Turmeric (*Curcuma longa*) is one of the herbal medicines used traditionally. It belongs to the *Zingiberaceae* family. Due to the existence of curcumin (a polyphenolic compound), the extracts of turmeric have shown antimicrobial and antioxidant activity. Therefore, the phenolic compound of curcumin is responsible for its antioxidant activities [48]. The phytochemical structures in turmeric include vitamin C, cineole, tumerone, borneol, zingiberene, d-sabinene, and d-phellandrene. Many types of chemical compounds are found in turmeric including sesquiterpene Ketones, monoterpenes, and sesquiterpene alcohols (e.g., zingeberene). Fresh turmeric contains zingiberene, while the most significant curcuminoid presented in turmeric is curcumin. Previous literature has reported that turmeric has an antimicrobial (antibacterial and antifungal) effect [49,140,141,142]. Curcumin is known for its inhibitory action on microorganisms such as *E. coli, S. aureus, Salmonella typhimurium*, and *Pseudomonas aeruginosa* [141,143]. In numerous literature works, turmeric’s extracts have been shown to have strong antioxidant properties. The main active compound of turmeric (curcumin) shows strong radical scavenger activity. It can scavenge RNS (reactive nitrogen species) and ROS such as superoxide radicals, alkoxy radicals, peroxyl radicals, hydrogen peroxide, singlet oxygen, peroxynitrite, hydroxyl radicals, and nitricoxide by three active sites through the electron transfer and hydrogen abstraction [144,145]. Curcumin also shows indirect antioxidant properties through a reduction process of numerous cytoprotective proteins including catalase, γ-glutamylcysteine ligase, glutathione S transferase, glutathione reductase, heme oxygenase 1, superoxide dismutase, and glutathione peroxidase [146,147]. Treatment with turmeric can reduce plasma malondialdehyde with increased glutathione reductase, glutathione peroxidase, catalase activity, and plasma albumin levels [148]. The aqueous and ethanol extracts of turmeric show significant antioxidant characteristics through the increase in antioxidant enzymes, scavenging different free radicals, and inhibiting lipid peroxidation [149]. Some in vivo studies on rats have demonstrated that turmeric inhibits hydrogen peroxide in cells by preventing lipid peroxidation [150,151]. The different extracts of turmeric, such as chloroform, *n*-butanol, ethyl acetate, and *n*-hexane, show strong antioxidant characteristics. Analyses have revealed a high correlation between the scavenging ability and the phenolic contents of these extracts [152]. Furthermore, the ethanolic extract shows high antiradical activity in rats through increased activity of antioxidant enzymes such as catalase, superoxide dismutase, and glutathione peroxidase [153]. The research shows antiviral activity of this plant against HIV virus [154,155].

### 2.7. Ginger

Ginger (underground rhizome of *Zingiber officinale roscoe* and herbaceous perennial plants) is one of the essential herbal medicinal plants from the *Zingiberaceae* family [156]. Ginger (the rhizome of *Zingiber officinale*) is native to Asia and has been used as a medicine for more than two thousand years around the world [157,158,159,160]. Ginger contains polyphenol components, including phenolic acids, gingerols, paradols, and shogaols. These principal components are responsible for its biological properties such as antioxidant, antidiabetic, antimicrobial, renoprotective, antihypertensive, antiulcer, anti-inflammatory, cardiovascular, analgesic, and gastrointestinal activities [50,51,161,162,163]. The antioxidant activity of ginger is related to the chemical compounds present in ginger such as zingiberene, zingerone, shogaols, and gingerols [164]. Some studies have analyzed the antioxidant activities of ginger and its components in numerous in vivo and in vitro lab experiments. Some researchers have demonstrated the potential antioxidant properties of ginger extract [165,166,167]. One study on rats has shown that ginger extract have antioxidant effect [168].

This herbal medicine also affects insulin secretion, insulin action, or both [169,170,171]. It can affect type one and type two diabetes because it can inhibit the metabolism of carbohydrates and lipids [172,173,174]. Extract of ginger shows high antimicrobial activity against dissimilar strains of bacteria such as *S. aureus*, *E. coli*, and *Salmonella typhi* [49,175]. Ginger has been used in several countries as a one of the most significant anticancer medicines based on the exogenous antioxidant activity of this plant. Therefore, it can be used as a treatment of diseases caused by free radicals [176]. Furthermore, the safety of using ginger as an antibiotic medicine has been investigated. In general, ginger has been known as a safe herbal medicine with pharmacological activity [177]. The antiviral activity of this plant has been demonstrated against influenza virus [178].

### 2.8. Thyme

Thyme (*Thymus vulgaris*) is one of the active antimicrobial herbal medicine plants which belong to the *Lamiaceae* family. It is more active against different bacteria and can inhibit the growth of bacteria such as *Lactobacillus plantarum*, *Brochothrix thermosphacta*, and *Brevibacterium* linens. This potent antimicrobial ability is related to the presence of high concentrations of carvacrol, thymol, and phenols in the extracts and essential oils of thyme [53,179]. Thyme extracts and essential oil have demonstrated a long list of medicinal properties, such as antibacterial, antioxidant, antitussive, spasmolytic, anticancer, and anti-inflammatory characteristics [52]. The extract of this plant has been traditionally used as an antitumor medicine due to its antioxidant property [180]. Numerous studies in the literature have demonstrated that the higher phenolic content of thyme is responsible for the high radical scavenging and antioxidant properties of this medicinal plant [181,182,183,184]. The structural variability of extracts and oils of thyme has been the subject of numerous studies [185]. Some studies on the antimicrobial activity of thyme plants have evaluated the composition of thyme extracts and oil influenced by growing conditions, the genotype, and ontogenic development [186,187]. The antimicrobial ability of thyme has been reported against *Pseudomonas aeruginosa*, *S. aureus*, *Klebsiella pneumonia*, *E. coli*, and *Bacillus* [188]. The antiviral activity of thyme has been reported against herpes simplex virus type 1 [189].

### 2.9. Pennyroyal

Pennyroyal (*Mentha pulegium*) is one of the aromatic herbs belonging to the *Lamiaceae* family. This herb is native to Europe, Asia, and Africa [190]. Based on the high antioxidant and antimicrobial ability of this plant, its extracts and essential oils have been traditionally used in medicine, especially for skin diseases, aromatherapy, and cosmetics [191,192]. Numerous studies have demonstrated the bioactive properties of this plant in different countries around the world, including Portugal, Turkey, Iran, and Greece, by focusing on the chemical composition of pennyroyal [192,193,194,195,196]. In the last decade, some researchers have demonstrated the antioxidant properties of pennyroyal extracts and essential oil by focusing on the chemical composition of this plant [197]. Phenols are the most bioactive component of the extracts and essential oil of pennyroyal. This organic compound has been shown to contain an OH functional group that is bound directly to the aromatic ring, and the hydrogen atom of the OH functional group can snare peroxyl radicals. Therefore, it can prevent the oxidation of other compounds [192,198]. Finally, the presence of phenol compounds is the reason for the antioxidant characteristic of this herbal medicinal plant [199]. Some researchers have studied the relationship between concentrations of the extract and essential oil of pennyroyal and their antioxidant activity. These results have shown a direct relation between the antioxidant activity and phenol content. The essential oil of this plant has also been shown to possess high antibacterial activity [200]. The antimicrobial ability of this plant is related to the presence of neo-menthol, pulegone, and menthone. Most studies have shown a strong antimicrobial ability of pennyroyal against different bacteria including *E. coli*, *S. typhimurim*, *Yersinia enterocolitica*, *Bacillus cereus*, *Listeria monocytogenes*, *Staphylococcus aureus*, *Clostridium perfrigens*, *Helicobacter pylori*, *Brochothrix thermosphacta*, *Pseudomonas aeruginosa*, and *Klebsiella*. The extract and essential oils of this plant pass through the cell wall of bacteria by damaging the membrane. This then results in disruption of the structure of polysaccharides, fatty acids, and phospholipids layers by an oxidation reaction [55,201]. This herbal medicine shows high inhibitory activity against herpes virus and influenza virus [202].

### 2.10. Fennel

Fennel (*Foeniculum vulgare*) is one of the herbal medicinal plants belonging to the *Apiaceae* family. Its native habitats include shores of Mediterranean Sea. There are some studies on the radical scavenging activity of fennel [203]. These studies have revealed that the antioxidant ability of this plant is due to the presence of high phenolic content in its extracts. Fennel has been shown to have high antioxidant ability. The antioxidant ability of the extract of this plant is due to numerous antioxidant processes such as free radical scavenging, superoxide anion radical scavenging, total antioxidant, and hydrogen peroxide scavenging [204,205]. The strong antioxidant characteristics of ethanol extracts and essential oil of this plant have been demonstrated by in vitro studies [206]. The hydro-ethanolic extracts of this plant have shown to possess free radical scavenging characteristics directly proportional to the content of phenolic compounds of fennel extract. The extracts and essential oil of this plant have been demonstrated to have significant antioxidant, antimicrobial, and anti-inflammatory properties [56]. The antimicrobial property of the essential oil (EO) and extract of fennel has been proven using the disk diffusion method [207]. Fennel extracts and essential oils have demonstrated high inhibitory activity against *Bacillus megaterium, Eschericha coli, Bacillus pumilus, S. aureus, Pseudomonas putida, Pseudomonas syringae, Salmonella typhi, Bacillus cereus, Micrococcus luteus, Klebsiella pneumonia* and *Bacillus subtili* [205,208]. The inhibitory ability of fennel also depends on its dosage. Consequently, fennel extract and oils could be a biosource of medicinal materials needed for the manufacturing of novel antimicrobial agents [209]. Fennel has been shown to be inhibitory against influenza A virus [210].

### 2.11. Chamomile

Chamomile (*Matricaria chamomilla*) is one of the traditional herbal medicines. This plant is part of the *Asteraceae* family and is still used in different medical applications, such as pharmaceutical and cosmetic industries [205,211]. Chamomile has a strong antimicrobial and antioxidant ability [212]. Various researchers have demonstrated the high antimicrobial ability of the extract and essential oil (EO) of this plant against various bacteria (Gram-positive and Gram-negative) including *E. coli, Salmonella thyphimurium, S. aureus*, and *Bacillus*. The high antimicrobial ability of this plant is also due to the high contents of phenolic compounds. Chamomile contains flavonoids, terpenoids, phenolic compounds, apigenin, and matricin [58,205,213,214,215]. The presence of flavonoids in the extract of this plant is the reason for its antioxidant characteristic. Chamomile shows various pharmacological activities including antioxidative, antibacterial, anti-inflammatory, antifungal, analgesic, anticancer, anti-hypoglycemic, anti-stress, antihypertensive, and hepatoprotective properties [57,58,216,217]. This plant shows antiviral activity against HSV-2 [218].

### 2.12. Mint

Mint (*Mentha*) is one of the aromatic perennial herbs belonging to the *Lamiaceae* family [219]. It has been used for various applications, such as pharmaceuticals and cosmetics applications [220]. The EO and aqueous extracts of mint potentially have antioxidant properties due to the existence of phenolic compounds [59,221,222,223]. Mint essential oil has been shown to be an effective alternative short-term treatment of irritable bowel syndrome in humans, due to its anti-inflammatory abilities [224]. The antioxidant activity of this plant exclusively relies on its chemical composition and can prevent oxidative stress at the cellular level or in a living organism. Other studies have reported the use of mint extract as an antioxidant and antimicrobial bioactive natural extract [225,226,227]. Numerous studies have revealed the inhibitory ability of this plant depending on the type of bacteria and its strong antimicrobial ability against Gram-positive bacteria, especially *S. aureus*. Other studies have reported that the antimicrobial effect of this plant with different oil concentrations. Mint oil shows strong antimicrobial ability against different bacteria including *S. aureus, S. epidermidis, E. coli, Bacillus cereus, Enterococcus faecalis*, and *Cronobacter sakazakii* [228,229]. This herbal medicine shows inhibitory activity against HSV-1 and HIV viruses [230].

### 2.13. Burdock

Burdock (*Arctium lappa*) is a popular traditional medical plant from Asia, America, and Europe belonging to the *Asteraceae* family. Based on the phenolic-rich compounds present in the extract, burdock has antimicrobial and antioxidant capabilities, as well as other biochemical activities including antipyresis and detoxifying [231,232,233,234,235]. Therefore, this plant could be used as an antioxidant, antimicrobial, and anti-inflammatory agent [60,236]. Studies have reported the antioxidant activity of burdock [237,238,239,240]. There is also information on the antimicrobial ability of burdock against different bacteria (Gram-negative and Gram-positive) including *S. aureus*, *S. epidermidis*, *Proteus mirabilis*, *Enterococcus faecalis*, *B. cereus* (*Bacillus cereus*), and *E. coli* because this plant is rich in phenolics [241,242,243]. In general, the extracts of burdock have demonstrated high antimicrobial activities and antioxidant capacity. The antioxidant and antimicrobial abilities of burdock extracts are due to the combined action of caffeic acid, rutin, o-hydrobenzoic acid, chlorogenic acid, and *p*-coumaric acid present in these extracts. Burdock leaves show high antimicrobial activities. The results of the previous studies have also conclusively revealed the antioxidant activities of the leaves of burdock [61,234]. The presence of phenolic-rich compounds in burdock is a potential source for its ability to inhibit oxidation activity [239]. This herbal medicine shows antiviral activity against herpesvirus (HSV-1, HSV-2), HIV, and adenovirus (ADV-3, ADV-11) [231].

### 2.14. Eucalyptus

Eucalyptus (*Eucalyptus*) is a member of the *Myrtaceae* family. It is called the fever tree based on its strong antimicrobial ability. This herbal medicine is native to the Mediterranean, Australia, and Tasmania area and it has been used as traditional medication for the treatment of numerous diseases including diabetes, pulmonary tuberculosis, bacteria and fungal infections, and influenza [244,245,246,247,248,249]. The medical applications of eucalyptus are based on the high antioxidant and antimicrobial abilities of its essential oil [62]. High concentrations of several polyphenolic compounds including flavonols, hydroxybenzoic acids, and hydrolyzable tannins have been found in the extract of eucalyptus [63]. These compounds are the reason for the high antimicrobial and antioxidant activity of eucalyptus. Recent studies have revealed the strong antibacterial ability of eucalyptus against *S. aureus*, *Listeria monocytogenes*, *Bacillus*, *Klebsiella pneumoniae*, *Enterococcus faecalis*, *Pseudomonas aeruginosa*, *Salmonella Enteritidis*, and *Escherichia coli*. These results suggest that eucalyptus has high antimicrobial and antioxidant abilities. The antioxidant ability of this herbal medicine is due to the presence of polyphenol, oenothein B, gallic acid, ellagic acid, flavonoids, and hydrolyzable tannin dimer in its extract. Eucalyptus extract also contains kaempferol-3-O-β-D-glucuronides and quercetin [250,251,252,253]. The presence of flavonoids also enhances the chances of having antioxidant ability in the extracts and essential oil of eucalyptus. Studies have reported that the radical scavenging ability of the eucalyptus essential oil increases by increasing the concentration of this herbal medicine plant [254]. This plant shows antiviral activity against herpes simplex virus (HSV) and influenza virus [255,256].

### 2.15. Primrose

Primrose (*genus Oenothera*) is a member of the *Onagraceae* family. Primrose shows numerous pharmacological effects including antimicrobial and antioxidant properties. This herbal medicine has been traditionally used for skin treatment [64,257]. The oils and extracts of primrose may also be used as natural antioxidants. The most important component of primrose includes various phenolic compounds which have been shown to be responsible for the antioxidant and free radical scavenging ability of primrose [258,259]. Numerous researchers have reported that the antioxidant activity of this plant is due to its phenolic components. Another group of chemical compounds present in primrose is triterpenoids. The antioxidant ability of primrose ethanolic extracts has been demonstrated in the literature [64,260,261]. It has been reported that the lipophilic triterpenoid esters present in essential primrose oil are the reason for the effective antioxidant activity of this plant [262,263]. Methanolic extracts of primrose were also demonstrated to possess potential antioxidant ability [264]. The radical scavenging ability of primrose was revealed against 2, 2-azino-bis (3-ethylbenzothiazoline-6-sulphonic acid). It was demonstrated that the alcoholic extract exhibits strong antioxidant ability [64]. The antioxidant ability of primrose was also demonstrated against DPPH, which was also revealed to possess strong antioxidant ability [265]. The oil of primrose has been used for the treatment of MS disease due to its immune-modulating and anti-inflammatory effects. Based on the concentration of primrose, it can show different inhibitory effects against various bacteria (Gram-positive and Gram-negative) [266,267,268]. The EO of primrose contains gamma linolenic acid (GLA). GLA has a strong ability in treating diabetes [269,270]. Primrose contains some alcohols, sterols, hydrocarbons, and tocopherols [65]. Sterols have been used for their antitumor properties and their cholesterol-lowering effects. Phytosterols present in primrose have also been shown to have antimicrobial and anti-inflammatory abilities [65]. Previous studies have demonstrated the high antimicrobial ability of the extracts and essential oil of primrose against different microorganisms such as *S. aureus*, and *E. coli* [267,271,272]. Recently, the methanolic and aqueous extracts of primrose have been shown to possess a high antibacterial effect against enterobacteria [273]. In vivo experiments have shown that primrose extract has high inhibitory effects on dental cavities caused by *S. mutans* in rats [274]. This plant shows antiviral activity against varicella virus, Epstein–Barr virus, and herpes simplex virus [64].

### 2.16. Lemon Balm

Lemon balm (*Melissa officinalis*) is one of the traditional herbal medicines belonging to the *Laminaceae* family. This herbal medicinal plant grows in North America, Europe, and Asia. Its use as a medicinal plant originated from Mediterranean countries [275]. The lemon balm has been used for several purposes such as medicine [276]. The extracts and EO of the lemon balm plant have some pharmacology effects including antimicrobial, anticancer, antibacterial, anti-cardiovascular diseases, antioxidant, anti-inflammatory, antispasmodic, and antiviral properties [66]. Some studies have demonstrated the effectiveness of lemon balm against different diseases such as HIV-1, cancer, and Alzheimer’s [277]. Lemon balm is a rich source of phenolic componds such as thymol and carvacrol which are the potential reason for the antibacterial and antioxidant activity of the lemon balm plant [67,278]. The antimicrobial characteristics of lemon balm have been used against Gram-negative bacteria including *E. coli*, *Salmonella typhi*, *Pseudomonas aeruginosa*, *Proteus*, and *Klebsiella* and Gram-positive bacteria including *S. aureus*, *Sarcina lutea*, *beta-hemolytic Streptococcus*, and *Bacillus cereus* [278,279,280]. The antimicrobial activities of lemon balm are mainly explained through the C15 and C10 terpenes with phenolic hydroxyl groups and aromatic rings, as well as other active terpenes, such as esters, aldehydes, and alcohols [278]. Moreover, these components have also demonstrated high antioxidant activity in lemon balm [281]. Some studies have reported the free radical scavenging ability of lemon balm together with positive results on lipid peroxidation. Other studies have revealed the antiviral activity of lemon balm’s essential oil [31,282,283].

### 2.17. Mallows

Mallow (*Malva sylvestris*) is another herbal medicinal plant which belongs to the *Malvaceae* family. This herb is native to Europe, America, and Asia [284]. The extract of this plant has high phenolic content and high antimicrobial properties. However, there is little clinical evidence regarding the use of mallow. The antibacterial effect of this herb has been reported against different bacteria (Gram-positive and Gram-negative) including *S. aureus*, *Bacillus cereus*, *E. coli*, *Klebsiella pneumoniae*, *Salmonella typhimurium*, *Listeria monocytogenes*, *Proteus vulgaris*, *Streptococcus pyogenes*, *Micrococcus luteus*, *Pseudomonas aeruginosa*, and *Mycobacterium smegmatis* [68,285,286,287]. In the last decade, mallow extract has been used for medicinal applications due to its excellent effect on the several systems of the body, including skin injuries and disorders, the muscular and skeletal systems, and the respiratory and digestive systems. Mallow has been shown to possess spasmolytic, lenitive, laxative, diuretic, demulcent, anti-diarrheal, bronchodilator, expectorant, and antitussive effects. It has high antimicrobial and antioxidant activity and is therefore highly recommended for skincare [288,289,290]. The antimicrobial and antioxidant activities of mallow are related to its chemical components including β-carotene, flavonoids, vitamin E, polyphenols, and vitamin C [69,291]. Polyphenols are the main reason for the excellent antioxidant and antimicrobial ability of mallow [292]. Due to the antioxidant effects of these phytochemicals, mallow is able to scavenge various free radicals, leading to the defence of biological molecules against oxidation. Therefore, the alcoholic and aqueous extracts of mallow have some pharmacological ability including antiviral, anti-inflammatory, antibacterial, and anti-cancerogenic effects [68,293].

### 2.18. Garlic

Garlic (*Allium sativum*) is an herbal medicine belonging to the *Amaryllidaceae* family [294]. Garlic is native to Central Asia, especially Iran [295]. Garlic has been demonstrated to possess biological activities including antioxidant, immunomodulatory activities, antidiabetic, anticancer, antibacterial, cardioprotective, and anti-inflammatory effects. The major components of garlic are phenolic, polysaccharides, and organosulfur contents. It also contains saponins, amino acid, flavonoids, vitamins A and C, B-complex vitamins, and minerals [70,296,297]. These chemical components are the reason for the biological activity of garlic. Garlic has been known as a natural antioxidant and can inhibit the harmful effects of free radicals in cells [298,299,300]. Antioxidant materials are naturally found in different plants and can neutralize free radicals through electron donation and by converting these harmful molecules to harmless products. Garlic is one of the traditional medicines with antimicrobial and antioxidant characteristics [301].

Numerous researchers have demonstrated the antioxidant property of garlic which is due to the presence of some chemical components including organosulfur, and phenolic compounds [70,302]. Garlic has a high content of phenolic compounds which are the reason for the high antioxidant activities of garlic [303]. Some researchers have indicated that the extract of garlic shows high scavenging of free radicals and therefore has powerful antioxidant ability [304,305]. Furthermore, other studies have reported that garlic shows antioxidant ability including one research which analyzed this ability using in vivo experiments [71,306]. Amino acids such as alliin represent one of the important components of garlic [307]. The alliinase enzyme can convert alliin to allicin which is one of the main components of garlic. This chemical compound is responsible for the antimicrobial activity of this herbal medicine [70,308]. Garlic was demonstrated to exhibit antibacterial activity against a varied range of different bacteria (Gram-positive and Gram-negative) such as *Klebsiella, Enterococcus faecalis, Pseudomonas, Salmonella typhi, Proteus, Staphylococcus aureus*, and *Escherichia coli* [309,310,311,312,313]. Some studies have also reported high antimicrobial and antioxidant activities of garlic based on the chemical reaction between allicin and thiol groups of various enzymes [314,315]. This plant also shows antiviral activity against influenza virus (H1N1) [316]. Table 2 demonstrates that some of the research has shown these herbal medicines including clove, portulaca, tribulus, eryngium, cinnamon, turmeric, ginger, thyme, pennyroyal, mint, fennel, chamomile, burdock, eucalyptus, primrose, lemon balm, mallows, and garlic to be potential antimicrobial agents. Table 3 and Table 4 show the antiviral and antioxidant activity of these materials.

## 3. Conclusions and the Future Outlook

**Figure 5:**
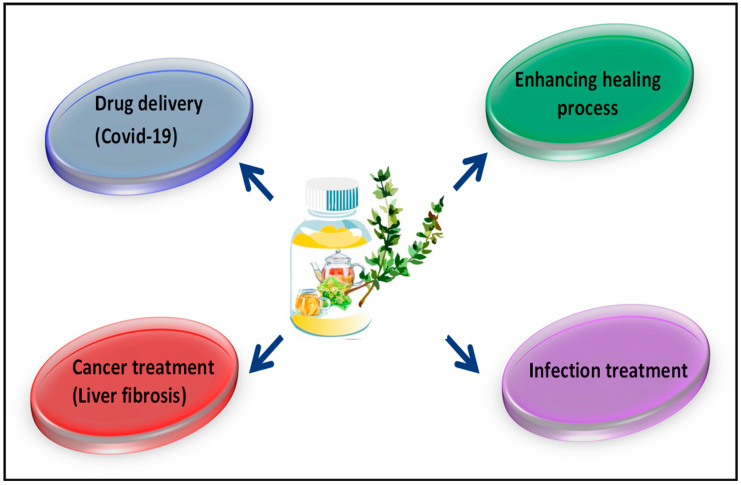
The future trend of herbal materials.

Many researchers have reported the biomedical applications of herbal medicines [346,347,348,349,350,351,352,353,354,355,356,357,358,359,360,361,362,363,364,365]. Antibiotics and other chemical antimicrobial agents including metal oxide and metal nanoparticles can inhibit bacteria growth but also lead to ROS generation and other side effects. On the other hand, herbal medicines such as clove, portulaca, tribulus, eryngium, cinnamon, turmeric, ginger, thyme, pennyroyal, mint, fennel, chamomile, burdock, eucalyptus, primrose, lemon balm, mallows, and garlic can eliminate bacteria by acting as a free radical scavenger. The antibacterial activity of these herbal medicines is different toward various kinds of bacteria and different kind of extract and essential oil of these herbal medicines [73,366]. In this light, it was exhibited [34,367] that the minimum inhibitory concentration (MIC) of these herbal medicines such as the essential oil of thyme towards various bacteria is in the range of 50–400 ppm for *E. coli*, *S. aureus*, *P. mirabilis*, *S. typhimurium*, *P. vulgaris*, *Y. enterocolitica*, *S. marcescens*, *B. licheniformis*, *P. putida*, *S. flava*, *P. fluorescens*, *L. innocua*, *Micrococcus spp*., *and B. thuringiensis* [34,367]. However, some other studies [368,369,370,371,372,373,374,375,376] evaluated positive aspects and restrictions of nanofibrous dressings with a great surface area and capacity to release antibiotics, herbal medicine, and antibacterial agents locally into the wound in order to the treatment of infection. It is worth noting that herbal medicines can act as direct antioxidants by blocking ROS generation and therefore can inhibit the planned cell death pathways. Thus, based on the lower concentration of ROS, the defense mechanism is started before the apoptosis pathway is prompted by glutamate. The presence of phenolic compounds in the extract or essential oil of these herbal medicines often demonstrates the potent antioxidant and antimicrobial properties. Therefore, herbal medicines are often preferred due to their less toxic and side effect-free nature compared to synthetic antimicrobial agents. Based on the high antimicrobial and antioxidant ability of these herbal medicines, they can be beneficial for healing all types of wounds. Herbal medicines also show a high antimicrobial potential at a lower price. Bioavailability is the primary essential measure for assessing the health benefits of herbal medicine for humans. In current years, the idea that natural remedies are more secure in comparison with prescription medications has acquired traction and contributed to a massive rise in phytopharmaceutical applications [377]. The bioavailability of these herbal medicinal products varies in blood, plasma, or tissue [378]. The bioavailability of these herbal medicinal products such as thyme [379], eucalyptus [380], turmeric [381], mint [378], garlic [382], ginger [377], cinnamon [377], and clove [377] was recorded in earlier research. These studies significantly lead to the precise scientific evaluation of the numerous remarks regarding the herbal products, which are progressively marketed with curative remarks throughout the world [378]. Therefore, the replacement of chemical antimicrobial agents with these herbal antimicrobial agents is a perfect solution due to their performance as an antimicrobial agent and also their radical scavenger ability. Herbal materials have created a novel exciting field in all sciences, especially in medicine, for future studies due to their unique properties. Their medicinal applications have already led to the development of new medical productions. Considering the decisive role of antimicrobial drugs in human life, these new fields in the drug industry have become increasingly more acceptable. However, designing new antimicrobial drugs free from side effects will not only create a new area of study but can also help meet the expanding human needs. Hence, this review suggests the use of herbal antimicrobial agents instead of synthetic ones due to their dual activity. In this paper, antioxidant, antimicrobial, and antiviral properties of individual herbs have been described comprehensively. Use of a combination of these herbs is also widespread in traditional medicine practice. Therefore, future research can focus on the characterization of the active component and the effect of herb–herb combinations for future therapeutic advancements and pharmaceutical product development. These materials shows high antiviral activity; therefore, these biomaterials are suggested for use against the COVID-19 virus. Therefore, these materials can be suggested to improve drug delivery (Covid-19), cancer therapy, infection, and acceleration of the healing rate, as shown in Figure 5.

## Figures and Tables

**Table 1:** A brief list of herbal antimicrobial agents and its medicinal applications and main biological compounds.

Herbal Materials	Medicinal Applications	Main Biological Compounds	Ref.
Clove	Antioxidant, antimicrobial, anti-inflammatory, anti-mutagenic, anti-allergic and anti-cancer.	Eugenol, eugenyl acetate, α-humulene, 2-heptanone, and β-caryophyllene	[38,39]
Portulaca	Antioxidant, antimicrobial, anti-inflammatory, anticancer, neuroprotective and antidiabetic.	Ascorbic acid, a-tocopherols, omega-3 fatty acids, apigenin, gallotannins, quercetin, and kaempferol	[40,41]
Tribulus	Antioxidant, antimicrobial, analgesic, anti-inflammatory and cardiovascular protective.	flavonoid, tannin and phenolic acids	[42,43]
Eryngium	Antioxidant, antimicrobial, anticancer, antidiabetic, antimalarial, anti-Alzheimer and anti-inflammatory.	Flavonoids, phenolic acids, and coumarins	[44]
Cinnamon	Antioxidant, antimicrobial, anti-inflammatory, anticancer, cholesterol-lowering, immunomodulatory and cardiovascular.	Cinnamaldehyde and eugenol	[45,46,47]
Turmeric	Antioxidant, antimicrobial, anti-inflammatory, anticancer, hypoglycemia and anticoagulant.	Vitamin-C, cineole, tumerone, borneol, zingiberene, d-sabinene, and d-phellandrene	[48,49]
Ginger	Antioxidant, antimicrobial, anti-diabetic, neuro- protective, analgesic, cardiovascular, gastrointestinal, anti-inflammatory, anticancer and antihypertensive.	Phenolic acids, gingerols, paradols and shogaols	[50,51]
Thyme	Antioxidant, antimicrobial, expectorant, spasmolytic, mucolytic and antitussive.	Carvacrol, thymol and phenols	[52,53]
Pennyroyal	Antioxidant, antimicrobial, anti-hepatic, Anti-genotoxic.	Neo-menthol, pulegone and menthone	[54,55]
Fennel	Antioxidant, antimicrobial and anti-inflammatory	Phenolic compounds	[56]
Chamomile	Antioxidant, antimicrobial, anti-inflammatory, anticancer, analgesic, anti-hypoglycemic, anti-stress and hepatoprotective.	Flavonoids, terpenoids, phenolic compounds, apigenin and matricin	[57,58]
Mint	Antioxidant, antimicrobial, anticancer and anti-inflammatory.	Phenolic compounds	[59]
Burdock	Antioxidant, antimicrobial, anti-proliferative and anti-inflammatory.	Caffeic acid, rutin, o-hydrobenzoic acid, chlorogenic acid, and p-coumaric acid	[60,61]
Eucalyptus	Antioxidant, antimicrobial anti-inflammatory and antipyretic.	Flavonols, hydroxybenzoic acids and hydrolyzable tannins	[62,63]
Primrose	Antioxidant, antimicrobial, anti-neuropathic, anti-inflammatory, anticancer and anti-ulcerogenic.	Phenolic acids, flavonoids, sterols, hydrocarbons, and tocopherols	[64,65]
Lemon balm	Antioxidant, antimicrobial, anti-stress, anti-Alzheimer, anti-inflammatory, anticancer, anti-cardiovascular and antispasmodic.	Phenolic componds such as thymol and carvacrol	[66,67]
Mallows	Antioxidant, antimicrobial, antinociceptive, anti-inflammatory, hepatoprotective and anticancer.	β-carotene, flavonoids, vitamin E, polyphenols, and vitamin C	[68,69]
Garlic	Antioxidant, antimicrobial, antidiabetic, anticancer, cardioprotective, anti-neurologica and anti-inflammatory.	Organosulfur such as Allicin, phenolic and polysaccharides compounds	[70,71]

**Table 2:** The herbal antimicrobial agents developed and their properties.

Herbal Biomaterials	Type	Minimum Inhibitory Concentration	Target of Bacteria	Result	Ref.
Clove	Essential oil	0.304 mg mL^−1^	*S. Typhimurium*, *S. aureus*, *L. monocytogenes*, and *E. coli*.	Good antibacterial efficiency against all tested bacteria	[73]
Portulaca	Extract	10 mg mL^−1^	*S. aureus*	High inhibitory activity against *S. aureus*	[86]
Tribulus	Metanolic extract	2 mg mL^−1^	*E. faecali, S. aureus, P. aeruginosa*, and *E. coli*	High effective antimicrobial activity against these bacteria	[317]
Eryngium	Essential oil	12.5 mg mL^−1^	*S. aureus*	Very interesting inhibitory activity against *S. aureus*	[117]
Cinnamon	Extract	62.5 mg mL^−1^	*S. aureus*	The good inhibitory effect against *S. aureus*	[318]
Turmeric	Essential oil	0.05 mg mL^−1^	*B. coagulans*, *S. aureus*, and *B. subtilis*	Maximum inhibitory activity was noted	[319]
Ginger	Essential oil	1 mg mL^−1^	*S. aureus*	Effective efficiency against *S. aureus*	[320]
Thyme	Essential oil	2.857 mg mL^−1^	*Klebsiella pneumoniae*	Good inhibitory activity	[321]
Pennyroyal	Alcoholic extract	400 mg mL^−1^	*Klebsiella* and *S. aureus*	Good inhibitory activity	[322]
Fennel	Essential oil	0.125 mg mL^−1^	*S. dysenteriae*	Effective inhibitory activity	[323]
Chamomile	Alcoholic extract	8 mg mL^−1^	*Klebsiella pneumoniae*	Effective antimicrobial activity	[324]
Mint	Extract	64 µg mL^−1^	*E. coli*	High inhibitory activity	[325]
Burdock	Extract	256 μg mL^−1^	*S. aureus*	The extract shows antibacterial activity against *S. aureus*	[241]
Eucalyptus	Essential oil	1000 μg mL^−1^	*S. aureus* and *E. coli*	Good bacteria inhibitory activty	[252]
Primrose	Methanol extract	0.07 mg L^−1^	*Listeria monocytogenes*	Highest inhibitory effect against *Listeria monocytogenes*	[326]
Lemon balm	Essential oils	0.25 mg mL^−1^	*Listeria strain*	Sensitive inhibitory effect against *Listeria strain*	[327]
Mallows	Ethanol extracts	0.4–0.8 mg mL^−1^	*S. pyogenes*, *P. vulgaris*, *S. aureus*, *and* *P. aeruginosa*	The extracts were active against *S. pyogenes*, *P. vulgaris*, *S. aureus*, *and* *P. aeruginosa*	[286]
Garlic	Extract	0.075 mg mL^−1^	*E. coli*	Significant antimicrobial efficiency against *E. coli*	[328]

**Table 3:** Antiviral activity of herbal materials and their virus target.

Herbal Biomaterials	Target of Virus	Ref.
Clove	Herpes simplex and hepatitis C	[329]
Portulaca	Influenza A viruses	[330]
Tribulus	Newcastle disease virus	[331]
Eryngium	HIV	[332]
Cinnamon	Influenza A virus, herpes simplex virus type 1 and 2	[333]
Turmeric	Epstein–Barr virus	[334]
Ginger	SARS-CoV-2 or COVID-19	[335]
Thyme	Influenza virus	[336]
Pennyroyal	HSV-1	[337]
Fennel	Para-influenza type 3 and Herpes simplex type 1	[338]
Chamomile	Influenza A virus	[339]
Mint	COVID-19	[340]
Burdock	COVID-19	[341]
Eucalyptus	COVID-19	[342]
Primrose	Hepatitis C virus (HCV)	[343]
Lemon balm	Influenza A virus (H9N2), SARS-CoV-2	[31,344]
Mallows	Influenza virus	[293]
Garlic	Influenza B and herpes simplex	[345]

**Table 4:** Antioxidant activity of herbal materials.

Herbal Biomaterials	Type of Free Radical	Type of Study	Medical Application	Ref.
Clove	DPPH	In vitro	Cancer therapy	[72]
Portulaca	DPPH	In vitro	Cancer therapy	[41]
Tribulus	DPPH, ABTS	In vitro	Infertility therapy	[99]
Eryngium	DPPH	In vitro	Cancer therapy	[111]
Cinnamon	DPPH and ABTS	In vitro	Antidiabetic	[137]
Turmeric	ROS	In vitro	Cancer therapy and antidiabetic	[144]
Ginger	DPPH	In vitro	Cancer therapy	[163]
Thyme	DPPH (2,2-diphenyl-1-picrylhydrazyl) radicals	In vitro	Cancer therapy	[181]
Pennyroyal	DPPH	In vitro	Cancer therapy	[196]
Fennel	DPPH (2,2-diphenyl-1-picrylhydrazyl) radicals	In vitro	Cancer therapy	[205]
Chamomile	DPPH (2,2-diphenyl-1-picrylhydrazyl) radicals	In vitro	Cancer therapy	[205]
Mint	ROS, 2,2-diphenyl-1-picrylhydrazyl, (DPPH) and 2,2′-azino-bis (3-ethylbenzothiazoline-6-sulphonic acid), (ABTS)	In vitro and in vivo	Cancer therapy	[227]
Burdock	ROS	In vivo	Antidiabetic	[238]
Eucalyptus	2,2-diphenyl-1-picrylhydrazyl, (DPPH)	In vitro	Antitumor	[250]
Primrose	Oxygen free radicals	In vivo	Anti-inflammatory	[261]
Lemon balm	ROS	In vitro	Infertility therapy	[283]
Mallows	DPPH	In vitro	Anti-inflammatory and anti-cancerogenic	[290]
Garlic	DPPH and ROS	In vitro andIn vivo	Cancer therapy and diabetes therapy	[301]
